# Effects of melanin from *Sepiella Maindroni* ink (MSMI) on the intestinal Microbiome of mice

**DOI:** 10.1186/s12866-017-1058-7

**Published:** 2017-07-03

**Authors:** Hui Dong, Weiwei Song, Chunlin Wang, Changkao Mu, Ronghua Li

**Affiliations:** 1 Collaborative Innovation Center for Zhejiang Marine High-efficiency and Healthy Aquaculture, Ningbo University, Ningbo, 315211 China; 2 Key Laboratory of Applied Marine Biotechnology, Ministry of Education, School of Marine Science, Ningbo University, Ningbo, 315211 China

**Keywords:** Melanin, *Sepiella Maindroni* Ink, High-throughput sequencing, Gut microbiota

## Abstract

**Background:**

By the search for new natural compounds with beneficial health effects, cephalopod ink has been considered as an attempt to develop new drugs and functional foods, which is an especially active field in Asia, where cephalopods are a major fishery catch, for which ink sacs are a bi-product and where homeopathic medicine has deep roots. There is a demand to evaluate the safety and influence to the organism. The specific composition and relative abundance of the gut microbiota, which is potentially a major modulator of host metabolism, drives the interaction between functional foods and host health. We explore the effects of melanin from *Sepiella Maindroni,* most common cuttlefish in China, on the intestinal microbiome of mice.

**Results:**

ICR mice were randomly divided four groups, which were normal group (S), low melanin dose group (D; 120 mg/kg), medium melanin dose group (Z; 240 mg/kg), and high melanin dose group (G; 480 mg/kg). Melanin was delivered for 28 consecutive days. Fecal samples were used to generate 7715 operational taxonomic units (OTUs) via high-throughput sequencing. There were significant shifts in relative abundance of the dominant taxa at the phylum, class, order, family, and genus levels following melanin treatment.

**Conclusions:**

MSMI had no significant effect on the structure of intestinal flora in mice. The main effect was in the proportion of dominant bacterial communities. The effect positively correlated with the dose. From a health point of view, the use of melanin does not cause intestinal flora disorder. Our results may have important implications for MSMI as functional food component and potential therapeutic for manipulating gut microbiota.

## Background

Cephalopods could protect themselves with the ejection of dark ink. The ink contains compounds that are capable of disrupting a predator’s chemical senses [1, 2]. There is a long history of using cephalopod ink as medicine and food. Mostly health benefits have been ascribed to cephalopod ink as a traditional medicine, both in Western culture (ancient Greece and Rome) and Eastern culture (China) [3]. Recent medical research proposed that cephalopod ink is a multifunctional bioactive marine drug and has the functions of antitumor, antioxidant, and anti-coagulant activities, as well as the capability to protection against testicular damage. Also, ink extract at the suitable concentrations can alleviate the immune response induced by cyclophosphamides in mice. Zhong et al. [4] studied the protective effects of squid ink extract on hemopoietic injuries induced by cyclophosphamide. Soliman et al. [5] reported that sepia ink could prevent kidney dysfunction induced by bile duct ligation and the invasive of pulmonary aspergillosis [6].

Cephalopod ink is a mixture of the co-secretions from the ink sac including glycosaminoglycan-like polysaccharides [7], a tyrosinase, and melanin. Among them, melanin a major component of ink has received the most interesting, which has been used in comparative studies of melanogenesis. The main monomer, the 5, 6-dihydroxyindole (DHI), and the 5, 6-dihydroxyinodole-2-carboxylic acid (DHICA) [8] are the build blocks of melanin. Melanin originates from different natural sources, and has been reported to be an excellent scavenger of free radicals; squid ink melanin is the most typically used melanin.

*Sepiella Maindroni* is one of the most popular dietary cephalopod species in the area of China coast. Data from our previous study [9] revealed that melanin from *Sepiella Maindroni* ink had anti-aging and immunomodulatory effects. The ink of squid has good application prospect in the field fo functional food. However, some scholars have found that cephalopod ink has antimicrobial properties against a diversity of organisms; meanwhile, less attention has been paid to the interactions between microbes and melanin. The gut microbiota is a functional organ; thus, clarifying the link between functional components and gut microbiota will benefit the theoretical basis for and utilization of functional foods [10]. The specific composition and relative abundance of the gut microbiota, which is potentially a major modulator of host metabolism, drives the interaction between functional foods and host health [11]. Therefore, understanding the impacts of melanin on the gut microbiota is essential.

We used high-throughput sequencing of variable regions of 16S ribosomal DNA to study the effects of MSMI on the microbiota. The degree of variation in the gut microbiota changed with oral melanin concentration. The composition and relative abundance of the gut microbiota after MSMI intervention were analyzed at the phylum, class, family, and genus levels. This study elucidates the structure of the gut flora under MSMI treatment. Furthermore, we provide a theoretical and applied basis for the development of MSMI as a functional food ingredient.

## Results

### Overall structural characteristics of the fecal bacteria

**Fig. 1:**
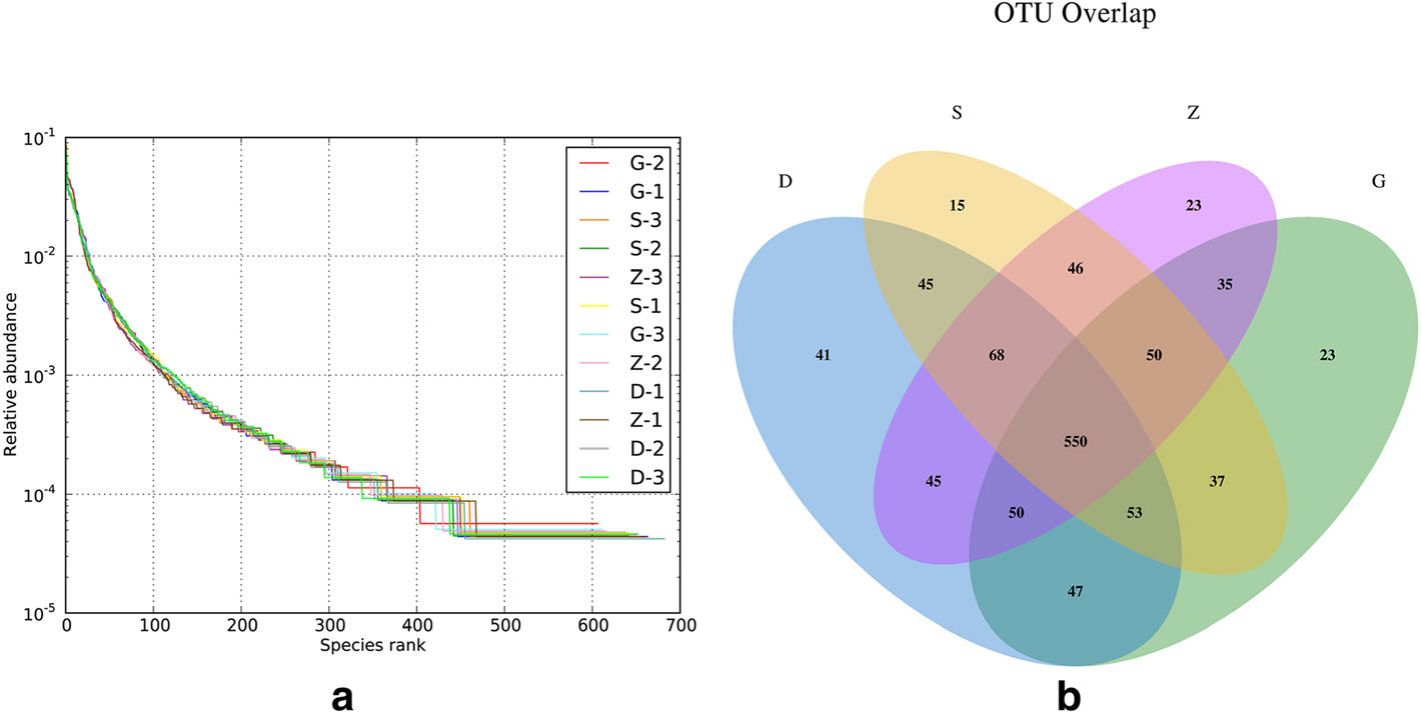
**a** Operational taxonomic units (OTUs) Rank Abundance. The X-axis indicates the OTU level according to abundance; the Y-Axis indicates the relative abundance of sequences contained in the OTU numbers; every *line* represents the OTU distribution of an individual sample. If the *curve* is more gentle, then the sample has a more uniform OTU distribution. **b** Core bacterial operational taxonomic units (OTUs) in mice from different treatment groups. The core community was determined based on OTUs detected in every fecal sample. The OTUs were assigned at a 97% sequences similarity threshold, and a Venn diagram was used to summarize the number of common OTUs amongst the four groups

Mice were divided into four groups, normal control group (S), low MSMI dose(D), medium MSMI dose(Z), and high MSMI dose(G), in which all of the males completed sampling after delivered for 28 consecutive days.There were 420,585 total reads, and 257,996 high quality reads generated from the fecal samples These were grouped into 7715 OTUs (Table 1) based on 97% similarity. The average number of OTUs was: 1937 for the saline group; 1984 for the low dose group; 1912 for the medium dose group, and; 1880 for the high dose group. Rarefaction curves indicated that OTUs’ were evenly distributed throughout the samples (Fig. 1a). The OTUs were classified into seven phyla, 12 classes, 11 orders, 21 families, and 28 genera. Among the four groups, about 550 OTUs were identified as core bacterial OTUs (Fig. 1b).

**Table 1:** Distribution of 16S OTUs amongst mice fed various concentrations of MSMI. The dietary treatment and number of OTUs obtained per fecal grab from each mouse is noted

Treatment	NO. 16S OTUs
S1	641
S2	654
S3	642
D1	652
D2	650
D3	682
Z1	638
Z2	615
Z3	661
G1	606
G2	611
G3	663

### Phylum-level comparison of fecal bacteria in mice

**Fig. 2:**
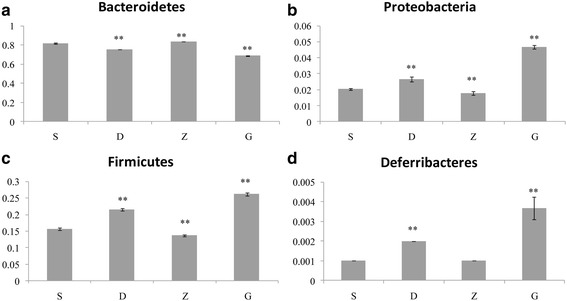
The four most abundant phylum of fecal microbiota in every group. The relative abundance of (**a**) Bacteroidetes, (**b**) Firmicutes, (**c**) Proteobacteria, and (**d**) Deferribacteres are expressed as the mean ± SEM, “* and **” indicate significance at “*p* < 0.05 and *p* < 0.01”, respectively compared with S group

The top four phyla were *Bacteroidetes, Firmicutes, Proteobacteria,* and *Deferribacteres* (Fig. 2). The groups receiving oral administration of MSMI had significantly different taxonomies from the saline (S) group. Overall bacterial abundance in MSMI groups was significantly decreased. (*p < 0.01*) (Table 2) However, there was a significant increase in the relative abundance of *Firmicutes, Proteobacteria,* and *Deferribacteres* after melanin treatment (*p < 0.01*). The Z group exhibited a significantly lower bacterial abundance than the D and G groups, which was closer to that of the S group. Particularly, the Z group had a similar abundance of *Deferribacteres* (*p* > 0.05). These results demonstrated that MSMI intervention impacts the fecal microbiota in mice to a certain degree. *Firmicutes* and *Bacteroidetes* are the dominant taxa in mice, and their ratio (F/B) in the S, D, Z and G groups was 0.191, 0.285, 0.163, and 0.381, respectively.

**Table 2:** Relative abundance of the top eight classes (%). The data are expressed as mean ± SEM. “*” and “**” indicate significance at “*p* < 0.05 and *p* < 0.01”, respectively when compared with the S group

Class	Phylum	S	D	Z	G
Bacteroidia	Bacteroidetes	0.817 ± 0.005	0.752 ± 0.002**	0.835 ± 0.002**	0.686 ± 0.005**
Clostridia	Firmicutes	0.155 ± 0.004	0.21 ± 0.004**	0.132 ± 0.004**	0.26 ± 0.005**
Epsilonproteobacteria	Proteobacteria	0.012 ± 0.000	0.009 ± 0.001**	0.006 ± 0.001**	0.035 ± 0.001**
Betaproteobacteria	Proteobacteria	0.003 ± 0.0006	0.011 ± 0.000**	0.008 ± 0.0006**	0.003 ± 0.0006**
Deltaproteobacteria	Proteobacteria	0.005 ± 0.0006	0.006 ± 0.000**	0.004 ± 0.0006**	0.008 ± 0.0006**
Verrucomicrobiae	Verrucomicrobia	0.003 ± 0.000	0.002 ± 0.000**	0.007 ± 0.0006**	0.000 ± 0.0000**
Deferribacteres	Deferribacteres	0.001 ± 0.000	0.002 ± 0.000**	0.001 ± 0.0006**	0.004 ± 0.0006**
Bacilli	Firmicutes	0.001 ± 0.000	0.002 ± 0.000**	0.004 ± 0.001**	0.001 ± 0.000**

### Class-level comparison of the fecal microbiota in the mice

**Fig. 3:**
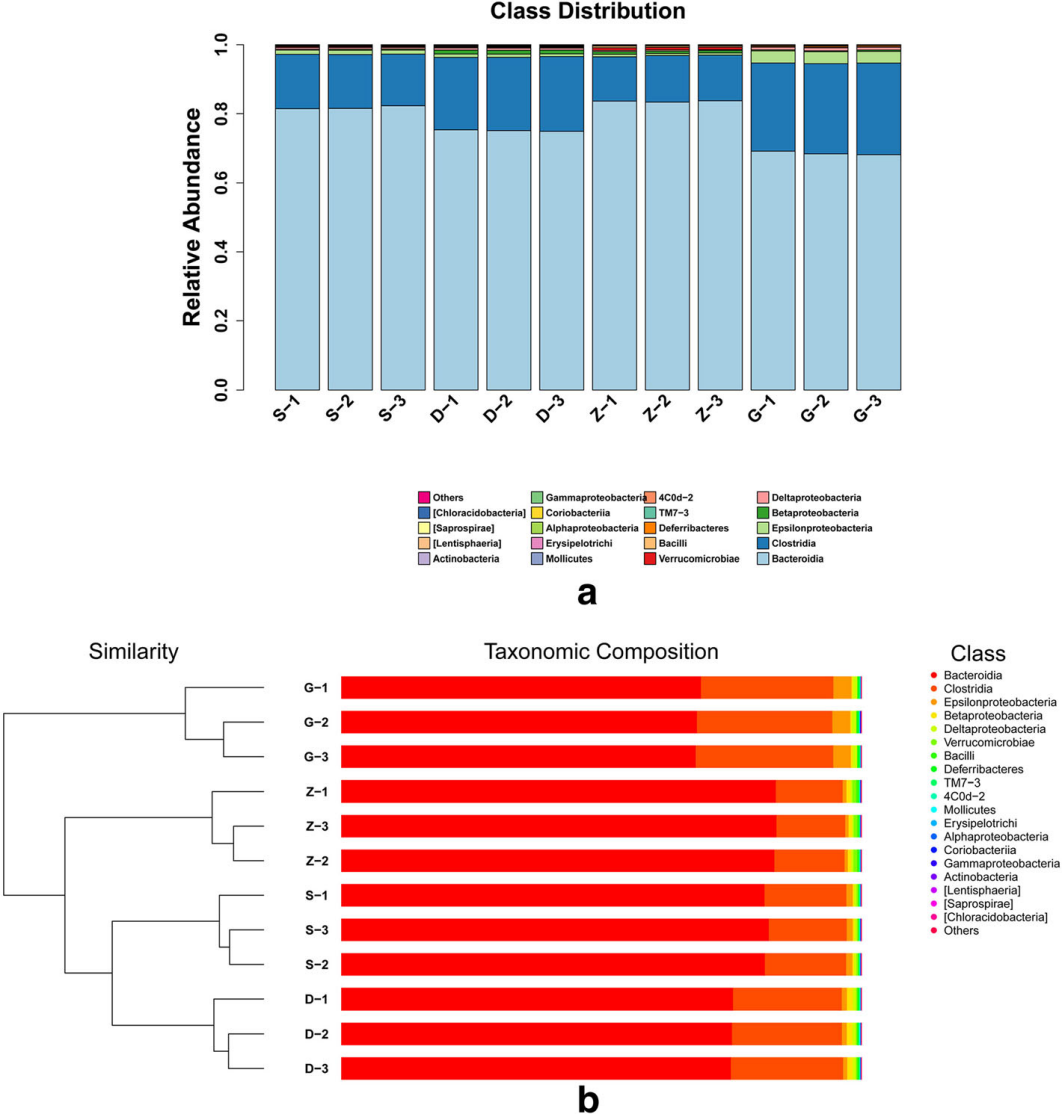
**a** *Bar chart* of taxonomic distribution at the class level. Different *colored bars* represent different bacterial classes. **b** Cluster histogram of samples at the class level

We calculated distribution at the level of class according to Bray-Curtis distance. The abundances of some *Firmicutes* (*Bacteroidetes, Clostridia,* and *Bacilli*), *Proteobacteria* (*Epsilonproteobacteria, Betaproteobacteria,* and *Deltaproteobacteria*), *Verrucomicrobia* (*Verrucomicrobiae)*, and *Deferribacteres* (*Deferribacteres*) had similar tendencies as the phyla (Fig. 3). The bacterial composition was dominated by two classes, *Bacteroidia* and *Clostridia*. The ratio of *Clostridia/Bacteroidia* (C/B) is consistent with the *Firmicutes/Bacteroidetes* ratio: 0.189, 0.282, 0.158, and 0.379 for the S, D, Z, and G groups, respectively.

### Family-level comparison of the fecal bacteria in the mice

**Fig. 4:**
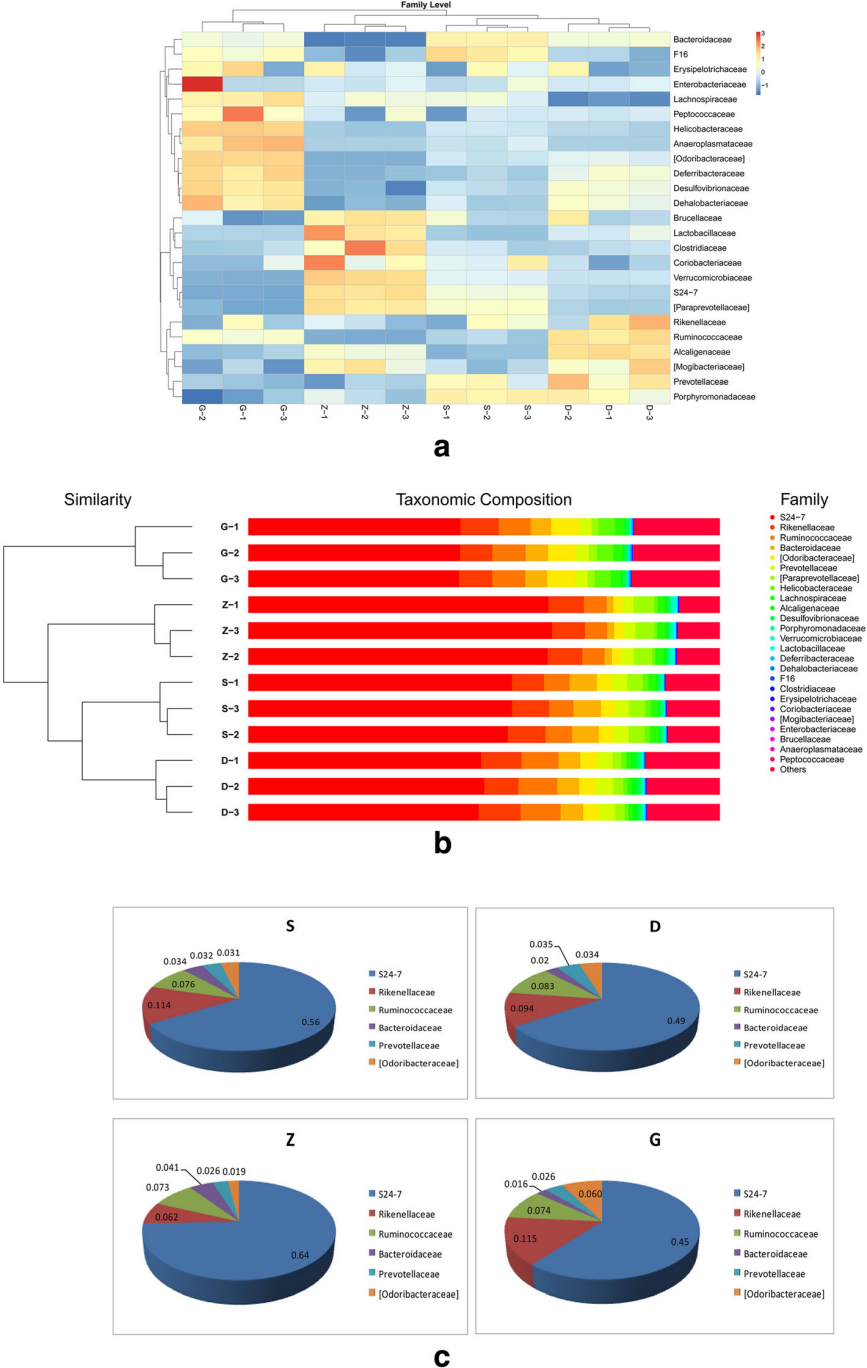
Beta diversity and relative abundance analysis at the family level. **a** Heat map of the 25 families with the highest frequency and relative abundance. **b** Hierarchically clustered heat map of the distribution of the top 25 bacterial families. **c** The top six most abundant bacterial groups. Different *color scales* represent different bacterial families

The 18 most abundant bacterial families were: *S24–7, Rikenellaceae, Ruminococcacea, Bacteroidaceae, [Odoribacteraceae], Prevotellaceae, [Paraprevotellaceae], Lachnospiraceae, Helicobacteraceae, Alcaligenaceae, Desulfovibrionaceae, Porphyromonadaceae, Verrucomicrobiaceae, Deferribacteraceae, Clostridiaceae, Dehalobacteriaceae, Erysipelotrichaceae,* and *F16* (Fig. 4). The six most abundant families in each group were: *S24–7, Rikenellaceae, Bacteroidaceae, Ruminococcaceae, Prevotellaceae,* and *[Odoribacteraceae]* in the normal group (S); *S24–7, Rikenellaceae, Ruminococcaceae, Bacteroidaceae, Prevotellaceae,* and *[Odoribacteraceae]* in the low dose group (D); *S24–7, Rikenellaceae, Ruminococcaceae, Prevotellaceae, [Odoribacteraceae],* and *Bacteroidaceae* in the medium dose group (Z), and; *S24–7*, *Rikenellaceae, Ruminococcaceae, [Odoribacteraceae], Bacteroidaceae,* and *Prevotellaceae* in the high dose group (G). Compared with S group, the G group had different taxonomy at the family level. On the whole, MSMI treatment caused alteration of intestinal flora; however, different doses had different effects.

### Response of the specific genera to the MSMI intervention

**Fig. 5:**
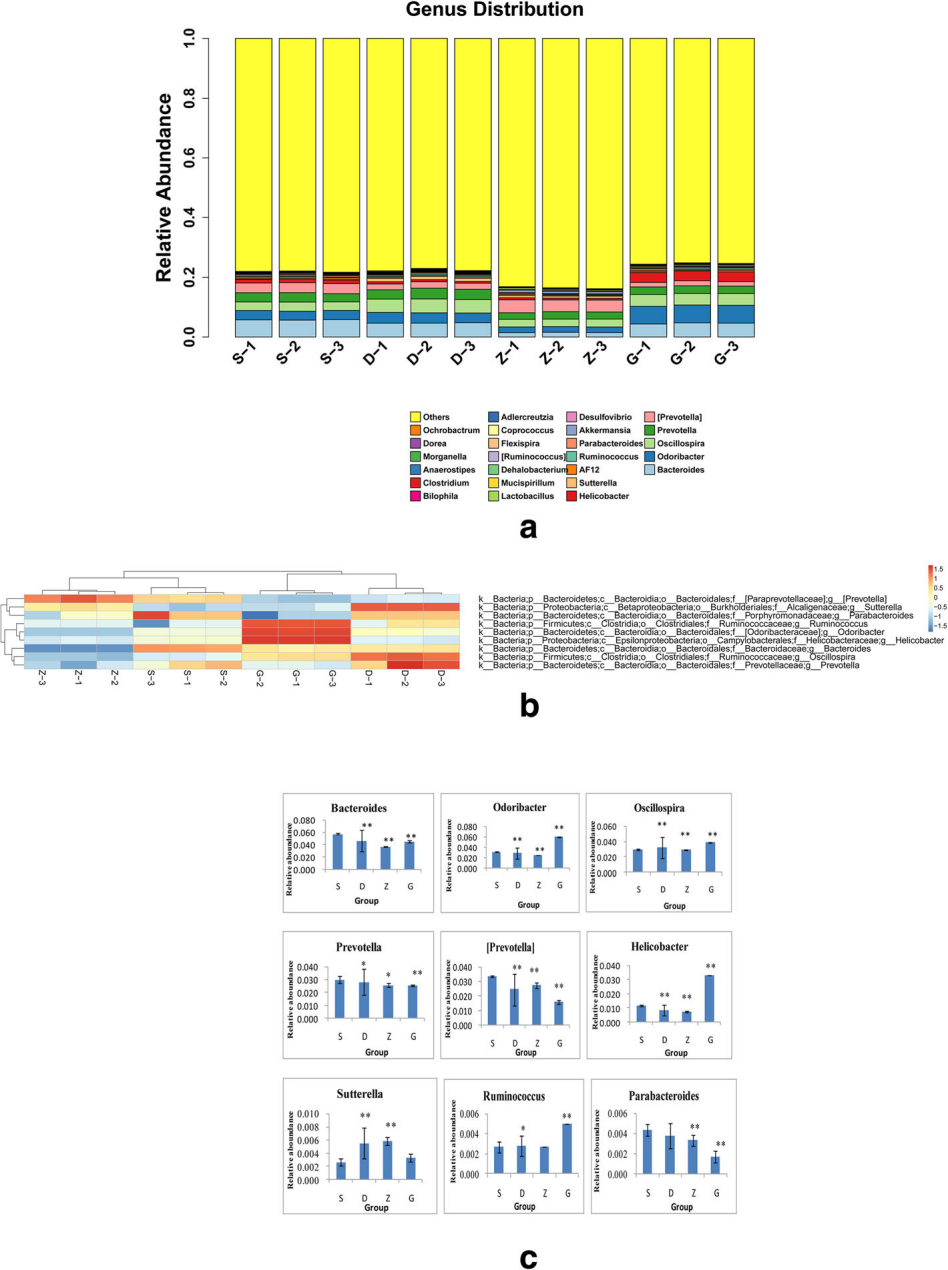
Beta diversity and relative abundance analysis at the genus level. **a** Hierarchically clustered heat map of the distribution of the 25 genera with the highest frequency and relative abundance. **b** Heat map of the nine genera with the highest frequency and relative abundance. **c** The relative abundance of *Bacteroides, Odoribacter, Oscillopsia, Prevotella, [Prevotella], Helicobacter, Sutterella, Ruminococcus,* and *Parabacteroides* expressed as the mean ± SEM. “*” and “**” indicate statistical significance at “*p* < 0.05 and *p* < 0.01”, respectively, compared with the S group

We assessed the 25 genera with high frequencies and relative abundances using a cluster bar chart (Fig. 5a). In general, samples from the medium and high MSMI dose groups differed in the relative abundance of these 25 genera. At the same time, there were nine genera detected at high levels in all four groups; these were further analyzed using a heat map (Fig. 5b). This showed that the relative abundance of these nine genera in the MSMI treatment groups had different branches compared with the S group. Moreover, the relative abundance of the nine genera (*Bacteroides, Odoribacter, Oscillopsia, Prevotella, [Prevotella], Helicobacter, Sutterella, Ruminococcus, Parabacteroides,* and *Lactobacillus*) that were altered by MSMI intervention have health and disease implications (Fig. 5c). As expected, we found statistically significant increases in the abundance of *Odoribacter, Oscillospira, Helicobacter* and *Ruminococcus*. Meanwhile, the relative of abundances some genera, such as *Bacteroides, Prevotella, [Prevotella], Sutterella,* and *Parabacteroides,* decreased with MSMI concentration.

### Bacterial diversity analysis of each group

**Fig. 6:**
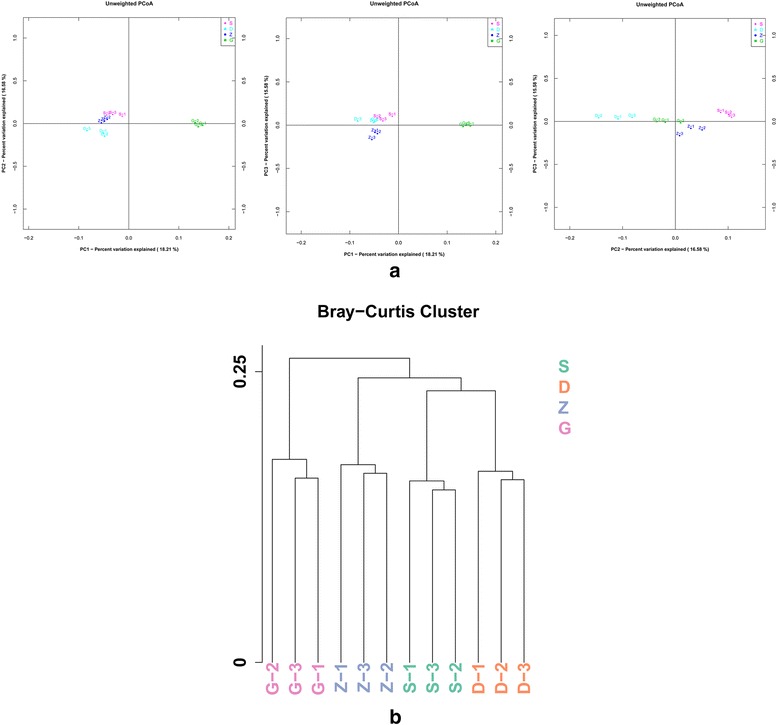
16SrRNA gene sequencing revealed developmental changes in bacterial communities. **a** Bacterial communities were clustered using PCoA of Unweighted UniFrac distance matrices. The percentages of variation shown by the plotted principle coordinates are indicated on the axes. **b** The Bray-Curtis distance was used to calculate the distance to each cluster for every sample. The X-axis represents the sample, and the Y-axis represents distance. The *branch plot* represents the sample distance, and different *colors* indicate the various groups

Differences and similarities between the microbial communities in the four groups were analyzed using weighted and unweighted Unifrac distances. Un-weighted Unifrac analysis showed that all of the mice had similar bacterial communities, as indicated by close clustering of samples on the two-dimensional PCoA plot (Fig. 6a). It indicates a possible developmental shift in the gut bacterial community among different MSMI treatment groups. On the whole, calculating the Bray-Cutis distance of the four groups revealed that the high MSMI dose group was further from the other groups (Fig. 6b). Additionally, Bray-Curtis Cluster analysis showed that the low MSMI dose group was more similar to the normal group (S).

## Discussion

The Recent medical research proposed that cephalopod ink be a multifunctional bioactive marine drug and has the functions of antitumor, antioxidant, and anti-coagulant activities, as well as the capability to protection against testicular damage [12, 13]. Dietary intervention influences gut microbiota composition and could be used as a therapeutic tool for alleviating unhealthy conditions triggered by microbial imbalances [14, 15]. We report taxonomic shifts at various levels in mice fed different doses of MSMI, which is crucial to assess the safety of MSMI as a functional food ingredient.

*Firmicutes* and *Bacteroidetes* are the dominant bacterial communities in the intestine, and the *Firmicutes/Bacteroidetes* ratio (F/B) in the S, D, Z and G groups were 0.191, 0.285, 0.163, and 0.381, respectively. Previous research has shown that the *Firmicutes: Bacteroides* ratio is positively correlated with the obese phenotype independent of diet [16]. Turnbaugh also compared the gut microbiota of lean mice and mice with diet-induced obesity and found that an increase in *Firmicutes* abundance was associated with diet-induce obesity [14]. In our study, MSMI increased the proportion of *Firmicutes* and decreased the *Bacteroidetes* proportion compared with the control. Maria et al. showed that the F/B ratio tends to grow with aging [17]; meanwhile, Guo et al. and Zhou found that preparation of water-soluble melanin from squid ink has an anti-oxidant activity which according to Scavenging free radicals [13] . The above results lead us to a concluded that *Firmicutes/Bacteroidetes* ratio increased is a positive effect.

*S24–7 (S), Rikenellaceae (Ri),* and *Ruminococcaceae (Ru)* were the three dominant families. They accounted for about 75% of relative abundance (Fig. 4c); however, their ratio (S:Ri:Ru) changed between groups: 7.4:1.5:1 for the S group, 5.9:1.1:1 for the D group, 8.8:0.8:1 for the Z group, and 6.1:1.6:1 for the G group. The abundance of *S24–7* and *Rikenellaceae* (both *Bacteroidetes*) were decreased in the D and G groups. It is consistent with prior studies showing that partial hepatectomies led to rapid changes in the gut microbiota, including an increased abundance of *S24–7* and *Rikenellaceae* (*Bacteroidetes*) and a decreased abundance of *Ruminococcaceae* (*Firmicutes*). Rogers et al. showed that *Rikenellaceae* (*Bacteroidetes*) were more abundant in anti-inflammatory drug users than in control patients [18]. It is evident from the forgoing testing that the change of gut microbiota positive protective effect.

MSMI altered taxonomy at the class level as well, including *Bacteroides, Odoribacter, Oscillospira, Prevotella, [Prevotella], Helicobacter, Sutterella, Ruminococcus,* and *Parabacteroides* (Fig. 5c). *Prevotella, Ruminococcus,* and *Oscillospira* thrive in a pro-inflammatory environment and are over-represented in inflammatory bowel diseases (IBD); moreover, these classes might even increase inflammation and incidences of colitis [19]. It suggests that appropriate MSMI concentrations may be capable of alleviating increases in *Prevotella, Ruminococcus,* and *Oscillospira* abundances have a real influence.

Altered gut microbiota caused by different MSMI doses were associated with the metabolism and pathology to provide new theoretical evidence for understanding the effect of melanin function. We also propose some new features and an appropriate MSMI concentration for use in functional foods.This study also explored the role of functional foods in shifting the host-gut microbial ecosystem. However, further studies are essential to verify the link between MSMI and microbial dysbiosis induced by inflammatory bowel disease, hepatopathy, and obesity. Further studies are also needed to determine if squid ink melanin can ameliorate the internal imbalance caused by D-galactose treatment.

## Conclusions

MSMI had no significant effect on the structure of intestinal flora in mice. The main effect was in the proportion of dominant bacterial communities. The effect positively correlated with the dose. From a health point of view, the use of melanin does not cause intestinal flora disorder. Our results may have important implications for MSMI as functional food component and potential therapeutic for manipulating gut microbiota.

## Methods

### Preparation of melanin from *Sepiella Maindroni* ink

Melanin from *Sepiella Maindroni* was prepared as previously reported [15]. Briefly, ink from fresh ink sacs was drained, mixed 20-fold with ultrapure water, and centrifuged (4000 g, 10 min, 4 °C). The precipitate was again mixed 20-fold with ultrapure water and centrifuged. The final melanin pellet was freeze-dried for approximately 48 h to get crude melanin. Highly purified melanin was obtained by incubating with 1.5% alcalde for four h at 50 °C, pH 10.1–10.3, and a substrate concentration of 2%. The resulting mixture was mixed six times with ultrapure water to remove the water-soluble impurities in ink. The final melanin pellet was lyophilized to get intact pure sepia ink melanin. The absorption spectrum is about 225.5 nm.

### Animals and treatment

Twenty male ICR mice (18.5 ± 2.6 g, 4 weeks old) were obtained from Animal Center of Zhe Jiang Province. During the experimental period, mice were housed in a room maintained under a 12 h light/dark cycle at 24 °C. Mice had free access to fresh water, as well as standard laboratory pellet chow. The chow met the requirements of the Chinese national standards.

Mice were randomly assigned to four groups of five mice each: normal control group (S), low MSMI dose, medium MSMI dose, and high MSMI dose, mice in every group were co-housed together with the same condition such as feeding the same batch of diet and water. For 28 days, the N group was given an oral administrated of normal saline once a day and the other three groups were given MSMI at either 120, 240, or 480 mg/kg/d by oral gavage. At the end of experimental period, mice were euthanized under chloroform vapor after being fasted overnight and cervically dislocated. All aspects of the experiment were conducted according to the guidelines provided by the Ethical Committee of Experimental Animal Care at Ningbo University (Ningbo, Zhejiang, China).

### Fecal bacteria collection and bacterial genomic DNA extraction

We fed the same strain, gender, and age of mice the same batch of diet to diminish the effects of genetic and environmental factors on the composition of bacteria. Cage effects were also considered [15], and the mice were co-housed together for 3 weeks to minimize the parent-cage effects before being randomly divided into different groups. Also, to avoid individual differences and obtain a clear understanding of the effect of dietary MSMI on intestinal microbiota, fecal samples were separately collected at day 28 (the last day of feeding period). Genomic DNA was extracted using a QIAamp DNA stool kit (Qiagen, Germantown, MD, USA) based on the manufacturer’s instructions.

### PCR amplification and pyrosequencing

DNA quantity, purity, and concentration were respectively tested by NanoPhotometer spectrophotometer and Qubit2.0 Fluorometer. PCR amplification was performed using the Fast-Start High Fidelity PCR System. To characterize the bacterial diversity present in the fecal samples, we used multiplex sequencing of the V3 and V4 hypervariable regions of the of 16S rDNA gene. The primer pair 314F (5′-CCTACGGGNGGCWGCAG-3′) and 805R (5′-GACTACHVGGGTATCTAATCC-3′), which product length was 465 bp, we used because it exhibits few biases and yields accurate phylogenetic and taxonomic information. The reverse primer contained a six bp-error correcting barcode unique to each sample. Target DNA sequence was amplified following a previously described protocol and constructed the library, at the same time, the specific index sequence was inserted. After the library is built, Qubit2.0 is used for initial quantification, and the library is diluted to 1 ng/ul. Using the Agilent 2100 to detect the insert size of the library, insert size matches the expected result Using Bio-RAD CFX 96 Fluorescence Quantitative PCR, Bio-RAD KIT iQ SYBR GRN QPCR, the effective concentration of the library to accurately quantify, to ensure the quality of the library. Detect qualified text Library, using Hiseq for sequencing, sequencing strategy for PE250.

### OTU clustering

The data were clustered at 97% identity against the Greengenes database (August 2013 release) using UCLUST, discarding reads that failed to match the reference sequences, which is referred to as a “closed reference” approach to clustering. Taxonomies came directly from the reference database based on the identity of the reference sequence clustered The 97% OTUs phylogenetic tree supplied with Greengenes was used for UniFrac metrics. The number of operational taxonomic units (OTUs) was used as a measure of microbiome richness, with OTUs being defined based on 4% divergence.

### Statistical analysis

16S rRNA raw data were analyzed with various statistical tests, including UniFrac procedures, hierarchal cluster analysis, distance-based redundancy analysis (db RDA), heatmap cluster analysis, and one-way ANOVA tests to determine the influence of dietary treatments on the microbial population.All the values in the tables and figures are mean ± standard error of the average. Statistical comparisons of the results were performed using an analysis of variance (ANOVA) in SPSS 13.0, *P* < 0.05 was considered statistically significant.

## Abbreviations

ANOVA: Analysis of variance
D: Low melanin dose group (120 mg/kg)
DHI: 5, 6-dihydroxyindole
DHICA: 5, 6-dihydroxyinodole-2-carboxylic acid
G: High melanin dose group (480 mg/kg)
IBD: Inflammatory bowel diseases
ICR: Institute of Cancer Research
MSMI: *Sepiella Maindroni* Ink
OTUs: Operational taxonomic units
S: Normal group
Z: Medium melanin dose group (240 mg/kg)
